# Biting midges (*Culicoides,* Diptera) transmit *Haemoproteus* parasites of owls: evidence from sporogony and molecular phylogeny

**DOI:** 10.1186/s13071-015-0910-6

**Published:** 2015-06-04

**Authors:** Dovilė Bukauskaitė, Rita Žiegytė, Vaidas Palinauskas, Tatjana A. Iezhova, Dimitar Dimitrov, Mikas Ilgūnas, Rasa Bernotienė, Mikhail Yu. Markovets, Gediminas Valkiūnas

**Affiliations:** Institute of Ecology, Nature Research Centre, Akademijos 2, Vilnius 21, LT-09412 Lithuania; Institute of Biodiversity and Ecosystem Research, Bulgarian Academy of Sciences, 2 Gagarin Street, Sofia, 1113 Bulgaria; Biological Station Rybachy of the Zoological Institute, Russian Academy of Sciences, Rybachy, 238535, Kaliningrad Region Russia

**Keywords:** Haemosporidian parasites, *Haemoproteus*, Owls, Sporogony, *Culicoides*, Vectors

## Abstract

**Background:**

*Haemoproteus* parasites are widespread, and several species cause diseases both in birds and blood-sucking insects. These pathogens are transmitted by dipterans belonging to the Ceratopogonidae and Hippoboscidae, however certain vector species remain unknown for the majority of *Haemoproteus* spp. Owls are often infected by *Haemoproteus* parasites, but experimental studies on vectors of these infections are lacking. The aim of this study was to investigate sporogonic development of two widespread *Haemoproteus* parasites of owls, *H. noctuae* and *H. syrnii* in experimentally infected biting midges *Culicoides impunctatus* and *Culicoides nubeculosus.* We also followed *in vitro* sporogonic development of these infections and determined their phylogenetic relationships with *Haemoproteus* spp., for which vectors have been identified.

**Methods:**

Wild-caught *C. impunctatus* and laboratory reared *C. nubeculosus* were infected experimentally by allowing them to take blood meals on one individual long-eared owl (*Asio otus*) and one tawny owl (*Strix aluco*) harbouring mature gametocytes of *H. noctuae* (lineage hCIRCUM01) and *H. syrnii* (hCULCIB01), respectively. The engorged insects were maintained in the laboratory at 16–18 °C, and dissected at intervals in order to follow the development of ookinetes, oocysts and sporozoites. We also observed *in vitro* development of sexual stages of both parasites by exposure of infected blood to air. The parasite lineages were determined by polymerase chain reaction-based methods. Bayesian phylogeny was constructed in order to determine the relationships of owl parasites with other avian *Haemoproteus* spp., for which vectors have been identified.

**Results:**

Both *H. noctuae* and *H. syrnii* completed sporogony in *C. nubeculosus,* and *H. noctuae* completed sporogony in *C. impunctatus*. Ookinetes, oocysts and sporozoites of these parasites were reported and described. Gametes and ookinetes of both species readily developed *in vitro*. In accordance with sporogony data, the phylogenetic analysis placed both parasite lineages in a clade of *Culicoides* spp.-transmitted avian *Haemoproteus (Parahaemoproteus)* spp.

**Conclusions:**

*Culicoides nubeculosus* and *C. impunctatus* are vectors of *H. noctuae* and *H. syrnii*. Phylogenies based on cytochrome *b* gene indicate parasite-vector relationships, and we recommend using them in predicting possible parasite-vector relationships and planning research on avian *Haemoproteus* spp. vectors in wildlife.

## Background

*Haemoproteus* parasites (Haemosporida, Haemoproteidae) are distributed worldwide, and several species cause diseases, sometimes severe, both in birds and blood-sucking insects [1–11]. This genus of haemosporidians contains subgenera *Parahaemoproteus* and *Haemoproteus,* where species are transmitted by blood-sucking insects of the Ceratopogonidae and Hippoboscidae, respectively [12–16]. In spite of numerous recent publications on avian haemosporidians [17–22], a few studies address *Haemoproteus* spp. vectors and transmission of these pathogens [14, 23–26], and experimental studies on these issues are few [5, 16, 27].

*Haemoproteus* parasites are widespread in owls worldwide, with prevalence of infection exceeding 50 % in many owl populations [28–31]. Pathogenicity of *Haemoproteus* infections in owls has been insufficiently investigated [5], but there is some evidence that these infections can be harmful. The co-infection of *Leucocytozoon danilewskyi* and *Haemoproteus noctuae* is likely responsible for mortality in snowy owl (*Nyctea scandiaca*), probably due to high parasitaemia [32]. However, vectors of this infection and other owl haemoproteids remain unidentified. A recent study based on observation of *Haemoproteus syrnii* in single naturally infected louse fly *Ornithomyia* sp., speculated that this widespread parasite of owls might be transmitted by hippoboscid flies [33]. However, traditional classifications placed *H. syrnii* and other haemoproteids of owls to the subgenus *Parahaemoproteus*, suggesting that they are transmitted by *Culicoides* spp. [5, 12]. Because experimental studies on vectors of owl *Haemoproteus* parasites are lacking, the aim of this study was to investigate sporogony of *H. noctuae* and *H. syrnii* in experimentally infected biting midges *Culicoides impunctatus* and *C. nubeculosus*. These biting midges are widespread in Europe and willingly take blood meals on birds [34]. Additionally, development of sexual stages of both these parasites was initiated *in vitro*. We also determined phylogenetic relationships among *Haemoproteus* spp., for which vectors have been identified, and discussed this information in regard to the possible perspectives in vector research of haemoproteids.

## Methods

### Study site, selection of experimental birds and collection of blood samples

Experiments were carried out at the Biological Station of the Zoological Institute of the Russian Academy of Sciences on the Curonian Spit in the Baltic Sea (55°05' N, 20°44' E) between 9 and 24 June in 2014.

In May 2014, one long-eared owl (*Asio otus*) and one common crossbill (*Loxia curvirostra*) were captured with big Rybachy traps [35] at the study site. Three tawny owls (*Strix aluco*) were examined for blood parasites in Kaliningrad Zoo, and we selected one bird for this experimental study. The experimental tawny owls originally came to the zoo from Kaliningrad District, Russia. All birds were kept outside in cages, which were protected from the penetration of biting midges and other blood-sucking insects by covers made of fine-mesh bolting silk. The birds were maintained at natural light–dark photoperiod (L/D) 17:7 h. The owls were fed with wild-caught mice and naturally died wild birds; they were held for approximately two weeks (the adaptation period) before they were used as donors of gametocytes in experiments. Both owls and the crossbill survived to the end of this study and were released after experimental work at the study site, which is a natural breeding area for these bird species.

Approximately 30 μl of blood was collected with microcapillaries by punching the brachial vein. One drop was used to make three blood films. The smears were air-dried within 5 – 15 s after their preparation, fixed in absolute methanol and stained with 10 % Giemsa solution, as described by Valkiūnas [5]. The blood smears were examined microscopically to identify parasite species and to control for the possible presence of natural co-infections with other haemosporidian parasites. Remaining blood was stored in non-lysis SET buffer (0.05 M Tris, 0.15 M NaCl, 0.5 M EDTA, pH 8.0) for molecular analysis. The samples were held at ambient temperature in the field and later at −20 °C in the laboratory.

Blood samples from donor birds were examined by polymerase chain reaction (PCR)-based methods. Amplifications were sequenced and cytochrome *b* (cyt *b*) lineages of *Haemoproteus* parasites were determined in the laboratory (see description below). One long-eared owl and one tawny owl with single infections of *H. noctuae* (cyt *b* lineage hCIRCUM01, gametocytaemia of 0.07 %) and *H. syrnii* (CULKIB01, gametocytaemia of 0.03 %), as determined both by microscopic examination and PCR-based testing, were used as donors of gametocytes to infect biting midges and to carry out *in vitro* experiments. Parasite species were identified according to Valkiūnas [5]. One uninfected juvenile common crossbill (*Loxia curvirostra*) was used to feed a control group of flies.

### Experimental design

Experimental infections of biting midges were performed in two ways. First, wild biting midges *C. impunctatus* were exposed to *Haemoproteus* infections near the Lake Chaika, located close to the village Rybachy, where density of *C. impunctatus* was high [36]. Second, we used the laboratory reared *C. nubeculosus* in experiments. Below, we describe these methods in more detail.

**Fig. 1:**
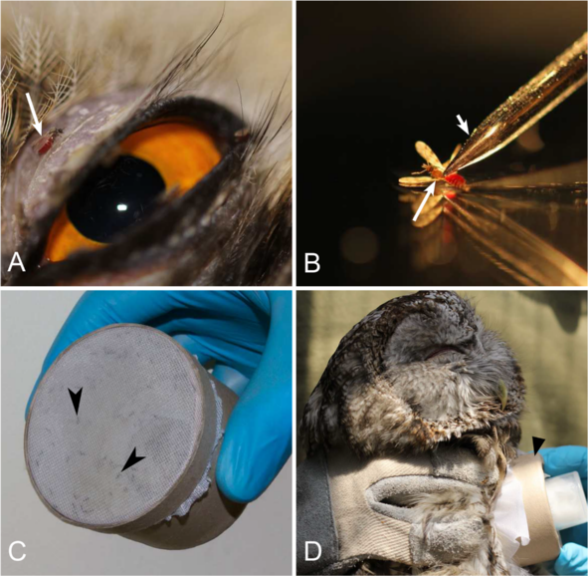
Experimental infection (**a**, **c**, **d**) and dissection (**b**) of the biting midges *Culicoides impunctatus* (**a**, **b**) and *Culicoides nubeculosus* (**c**, **d**) for detection of sporogonic stages of owl *Haemoproteus* spp. **a** – engorged biting midge *C. impunctatus* (long arrow) taking blood meal close to an eye of the long-eared owl *Asio otus*; **b** – dissection of *C. impunctatus*, short arrow indicates a dissecting needle; **c** – a hand-held cardboard box with a colony of *C. nubeculosus* inside, arrowheads indicate the midges sitting on fine-mesh bolting silk covering one side of the box; **d** – experimental infection of the colony of *C. nubeculosus* midges by means of adherence of a box with flies to skin of the tawny owl *Strix aluco*, triangle arrowhead indicates the box with flies

Wild-caught *C. impunctatus* biting midges were infected by allowing them to take blood meals on two infected owls and one uninfected crossbill (control), as described by Valkiūnas [5]. Briefly, the birds were held in the hands covered by rubber gloves. Biting midges were allowed to feed naturally on infected birds between 10 pm and 12 pm. The birds were exposed to bites for approximately 1.5–2 h depending on density of biting midges at the study site. This biting midge willingly takes blood meals on the feather-free region of body, and the insects were particularly often observed close to eyes (Fig. 1a). When several females began taking blood meals on a bird’s head, the head with feeding insects was carefully placed into an unzipped insect cage (approximately 12 × 12 × 12 cm) made of fine-mesh bolting silk. The engorged females fly off after the blood meals. The cage with engorged biting midges was then closed using a zipper. Up to 20 females were allowed to take blood meals on each bird daily. Cages with engorged flies were transported to the laboratory. To feed the biting midges, several pads of cotton-wool moistened with 10 % saccharose solution were placed on the top of each cage. Thirty-four infected *C. impunctatus* insects were used for dissection.

*Culicoides nubeculosus* biting midges were reared in the laboratory according to Boorman [37]. Each insect colony was maintained in small (approximately 5 cm in diameter) cardboard boxes covered by fine mesh bolting silk (Fig. 1c). The laboratory reared biting midges were delivered to the Biological Station and exposed by gentle pressing of a box with flies to a feather-free area on pectoral muscles of owls (Fig. 1d). Biting midges willingly took blood meals via fine-mesh bolting silk, and the majority of females were fully engorged approximately 30 min after the exposure. Twenty-four infected *C. nubeculosus* insects were used for dissection.

Both wild caught *C. impunctatus* and laboratory reared *C. nubeculosus* were held in the same room and conditions, i. e. 16–18 °C, 70 ± 5 % relative humidity and L/D photoperiod of 17:7 h. Biting midges were fed with 10 % saccharose solution; pads of cotton-wool were moistened in this solution and placed on the top of each insect cage daily.

In order to determine prevalence of possible natural *Haemoproteus* infection in wild-caught *C. nubeculosus*, 200 females were collected at the study site and tested by PCR-based methods (see description below). DNA was extracted from 40 pools of biting midges, each containing 5 flies.

### Dissection of biting midges and making preparations of parasites

Before dissection, biting midges were identified morphologically according to Gutsevich [38]. Insects were dissected at intervals in order to follow the development of ookinetes, oocysts and sporozoites. Engorged females were anesthetized by putting them into a tube with a cotton pad wetted in 96 % ethanol. Then, each insect was placed in a drop of 0.9 % normal saline. Midguts and salivary glands were extracted using dissecting needles (Fig. 1b), which were disinfected in fire to avoid contaminations after each dissection. We examined midgut contents for ookinetes 0.5–1 day post exposure (dpe), midgut wall for oocysts 3–8 dpe, and salivary glands for sporozoites 6–10 dpe.

Parasite preparations were prepared according to Valkiūnas [5]. Briefly, the midguts and salivary glands were extracted, gently crashed to prepare small smears, air dried, fixed with methanol and stained with Giemsa. Midgut preparations were stained in the same way as blood smears, while 4 % staining solution was used to stain preparations of salivary glands. Oocysts were first visualised by adding a minute drop of 2 % mercurochrome solution on freshly prepared midgut preparation, which was then covered with a coverslip. That simplifies the search of tiny *Parahaemoproteus* parasite oocysts. Oocyst-infected midguts were fixed in 10 % formalin solution for 24 h; formalin was then replaced with 70 % ethanol. After 6 h, the midgut preparations were washed with distilled water, stained with Ehrlich’s hematoxylin for 10 min, steeped in water containing a pinch of sodium bicarbonate and differentiated with acid ethanol both for 5 min and again steeped in water with sodium bicarbonate. Then, each preparation was dehydratated with 70 % and 96 % ethanol, cleared by putting a drop of clove oil and xylene over the preparation, and finally mounted in Canada balsam and covered with a cover slip. After dissection, all residual parts of insects were placed in 96 % ethanol to confirm the presence of parasite lineages by PCR-based methods (see description below).

### *In vitro* sporogonic development

Approximately 300 μl of blood was taken from the brachial vein of the infected owls. The blood was immediately placed in an Eppendorf tube and diluted with 3.7 % solution of sodium citrate (pH 8.5) in ratio 1 part of solution to 4 parts of blood. The work was performed at 20 °C ± 1 °C. To follow *in vitro* development of the parasites, smears were prepared 12 h after exposure to air. They were air-dried, fixed in methanol and stained with Giemsa, as described for blood films.

### Microscopic examinations of preparations and parasite morphology

**Fig. 2:**
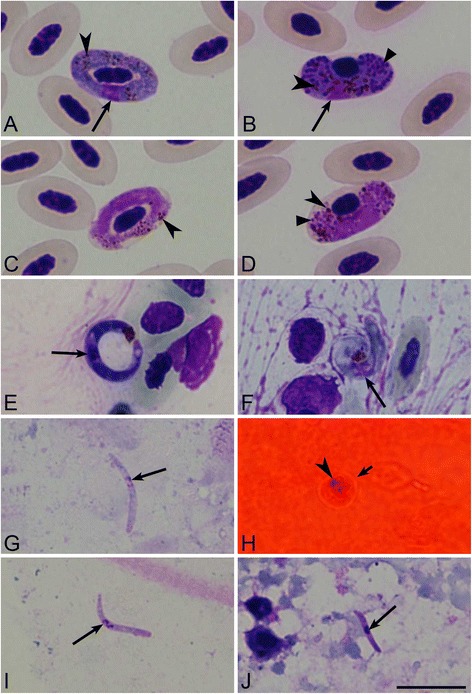
Mature gametocytes (**a**–**d**) and sporogonic stages (**e**–**j**) of *Haemoproteus noctuae* (lineage hCIRCUM01) in *Culicoides nubeculosus* (**e**, **i**) and *Culicoides impunctatus* (**g**), and *Haemoproteus syrnii* (hCULCIB01) in *C. nubeculosus* (**f**, **h**, **j**). Macrogametocytes (**a**, **b**) and microgametocytes (**c**, **d**) of *H. noctuae* (**a**, **c**) and *H. syrnii* (**b**, **d**) in peripheral blood of donor birds before experimental infection of biting midges. Ookinetes (**e**, **f**), oocyst (**h**) and sporozoites (**g**, **i**, **j**) are shown. Long simple arrows – nuclei of parasites, simple arrowheads – pigment granules, triangle arrowheads – volutin granules, short arrow – oocyst. Scale bar = 10 μm

All preparations were examined with Olympus BX-43 light microscope equipped with Olympus SZX2-FOF digital camera and imaging software QCapture Pro 6.0, Image-Pro plius (Tokyo, Japan). Blood films were examined for 15–20 min at low magnification (×400), and then at least 100 fields were studied at high magnification (×1000). Intensity of parasitaemia was estimated each day before exposure of biting midges; it was determined as a percentage by actual counting of the number of mature gametocytes (Fig. 2a–d) per 1000 red blood cells. All vector preparations were first examined at low magnification (×100, ×600) and then at high magnification (×1000). The statistical analyses were carried out using the “Statistica 7” package. Student’s *t*-test for independent samples was used to determine statistical significance between mean linear parameters of parasites. A *P* value of 0.05 or less was considered significant. Representative preparations of blood stages (accession nos. 418836–48838 NS) and vector stages *in vitro* (48840–48844 NS) and *in vivo* (48845–48856 NS) were deposited in Nature Research Centre, Vilnius, Lithuania. Two voucher preparations of blood stages were deposited in the Queensland Museum, Queensland, Australia (accessions G465772, G465773).

### Polymerase chain reaction and sequencing

Total DNA was extracted from all samples using ammonium acetate extraction method [39]. For genetic analysis, we used a nested PCR protocol [40, 41]. Amplification of the cyt *b* gene was done using two pairs of initial primers, HaemNFI and HaemNR3 for detection of *Haemoproteus*, *Plasmodium* and *Leucocytozoon* species. For the second PCR, we used primers HaemF and HaemR2, which are specific to *Haemoproteus* and *Plasmodium* parasites. All samples were evaluated by running 1.5 μl of PCR product on a 2 % agarose gel. One negative control (nuclease-free water) and one positive control (an infected sample, which was positive by microscopic examination of blood films) were used per every 14 samples. No cases of false positive or negative samples were reported.

To support species identification of wild-caught biting midges, we used the insect-specific primers LCO149 and HCO2198, which amplify a fragment of cytochrome oxidase subunit I of mitochondrial DNA [42].

Fragments of DNA from the PCR positive samples were sequenced from the 3′ and 5′ ends. The genetic analyzer “Basic Local Alignment Search Tool” (National Centre of Biotechnology Information website: http//www.ncbi.nlm.nih.gov/BLAST) was used to determine lineages of detected DNA sequences. Sequences were edited and aligned using BioEdit [43] and deposited in GenBank (accessions KP794611 and KP794612).

### Phylogenetic analysis

To determine phylogenetic relationships of *H. syrnii* and *H. noctuae* with other avian haemoproteids, for which vectors have been identified, we constructed a phylogenetic tree using 21 sequences of the mitochondrial cyt *b* gene of *Haemoproteus* spp. and three sequences of *Plasmodium* spp.; each sequence was of 479 bp. The tree was created using Bayesian phylogenetics as implemented in mrBayes version 3.1 [44]. Best-fit model of evolution (GTR + I + G) was selected by software Modeltest 3.7 [45]. We ran two independent analyses with a sample frequency of every 100th generation over 12 million generations. For construction of the majority consensus tree, 25 % of the initial trees in each run were discarded as burn in periods. We visualized the tree using the software Tree View 1.6.6. (available from <http://evolution.genetics.washington.edu/phylip/software.html>). The sequence divergence between the different lineages was calculated with the use of a Jukes-Cantor model of substitution, with all substitutions weighted equally, implemented in Molecular Evolutionary Genetics Analysis (MEGA) software, version 4.0 [46].

### Ethical statement

The experiments described herein comply with the current laws of Lithuania and Russia. Experimental procedures were approved by the International Research Co-operation Agreement between the Biological Station Rybachy of the Zoological Institute of the Russian Academy of Sciences and Institute of Ecology of Nature Research Centre (25-05-2010). Kaliningrad Zoo provided one naturally infected tawny owl for experimental research according to the ethical approval of 05-06-2014. All efforts were made to minimize handling time and potential suffering of birds. None of the experimental birds suffered apparent injury during experiments and were released after experiments.

## Results

Common crossbill used for control was parasite negative both by PCR and microscopic examination. Morphological examination and PCR-based testing showed that all experimental wild-caught flies belonged to *C. impunctatus*. According to PCR-based analysis, no natural infection was found in wild-caught biting midges*.* The lack of infected insects among wild-caught females is not surprising because 1) the natural infection of biting midges with hemoproteids usually is light (<1 %, see Valkiūnas [5]), and we carried out experiments in the beginning of the season of their activity when females likely took the first blood meal, so a probability to get natural infection was light. Parasites were also not detected in control flies both by PCR-based and microscopic examination methods.

In accordance to microscopic observation, the PCR and sequencing confirmed the presence of corresponding parasite lineages hCIRCUM01 and hCULCIB01 in experimentally infected biting midges.

### Sporogony of *H. noctuae* and *H. syrnii* in biting midges

Sporogonic development of the parasites was observed in all dissected infected insects. Ookinetes, oocysts and sporozoites of *H. noctuae* were seen both in experimentally infected wild-caught *C. impunctatus* and laboratory reared *C. nubeculosus* biting midges, indicating completed sporogony (Fig. 2e–j). *Haemoproteus syrnii* completed sporogony in *C. nubeculosus*, but only ookinetes and oocyst were found in *C. impunctatus*. Sporozoites of *H. syrnii* were not seen in salivary gland preparations of the latter biting midge probably due to the light infection. Additionally, only 2 exposed flies survived and were dissected 7 dpe, and sporozoites might not reach salivary glands or were few and difficult to find because of the small sample and short observation period.

Ookinetes of *H. noctuae* were seen 12 h post exposure both in C*. impunctatus* and *C. nubeculosus* (Fig. 2e). Ookinetes of *H. syrnii* were reported in *C. nubeculosus* 14 h post exposure (hpe) (Fig. 2f). Ookinetes of both species were elongated worm-like bodies possessing prominent, slightly off centre located nuclei and large ‘vacuoles’. Residual pigment granules were clamped at the distal ends of the parasites (Fig. 2e, f). In *C. impunctatus,* ookinetes of *H. noctuae* measured (*n* = 20) 17.0–24.5 (on average 20.7 ± 1.9) μm in length, 1.8–2.9 (2.4 ± 0.3) μm in width, 32.3–54.5 (41.2 ± 5.5) μm in area, and 3.1–8.0 (5.5 ± 1.6) μm in nucleus area. A few ookinetes of *H. syrnii* were seen, and they were not measured.

Oocysts of *H. noctuae* and *H. syrnii* were seen in fresh preparations both in *C. impunctatus* and *C. nubeculosus* biting midges 4–5 dpi, but only oocysts of *H. syrnii* (Fig. 2h) were observed and measured in permanent preparations. Oocysts of *Hamoproteus* parasites are tiny and difficult to observe and calculate on midguts; between 1 and 30 oocysts were observed in different oocyst preparations. Flattened oocysts of *H. syrnii* measured (*n* = 21) 4.0–8.1 (5.4 ± 0.9) μm in minimum diameter, 5.1–8.3 (6.1 ± 0.8) μm in maximum diameter, and 19.7–60.4 (27.7 ± 9.7) μm, in area.

Sporozoites of *H. noctuae* (Fig. 2g, i) and *H. syrnii* (Fig. 2j) were reported in salivary gland preparations 7–9 dpi, indicating that these biting midges are likely vectors of these parasites.

Morphologically identical sporozoites of *H. noctuae* developed both *C. impunctatus* and *C. nubeculosus*; they were fusiform bodies with off centre located nuclei and approximately equally pointed ends (Fig. 2g, i). There was no significant difference in the length, widths, area or area of nuclei in *H. noctuae* sporozoites (Table 1), which developed into different species of biting midges (*P* > 0.05 for all these features). Sporozoites of *H. syrnii* were also fusiform bodies with approximately equally pointed ends, but nuclei were located centrally (Fig. 2j). Additionally, sporozoites of *H. syrnii* were significantly shorter and smaller in area than those of *H. noctuae* (*P* < 0.05 for both these features). Based on these characteristics, sporozoites of *H. noctuae* and *H. syrnii* can be readily distinguished from each other (Table 1).

**Table 1:** Morphometry of sporozoites of two *Haemoproteus* species in the biting midges *Culicoides impunctatus* and *Culicoides nubeculosus*

Feature	Measurements^a^		
	*H. noctuae*		*H. syrnii*
	*C. nubeculosus*	*C. impunctatus*	*C. nubeculosus*
	*N* = 21	*N* = 16	*N* = 15
Length	9.3–13.2 (11.4 ± 1.0)	9.3–14.1 (12.3 ± 1.5)	6.3–9.4 (7.8 ± 0.9)
Width	1.0–1.4 (1.1 ± 0.1)	0.8–1.5 (1.2 ± 0.1)	0.9–1.5 (1.2 ± 0.2)
Area	8.2–13.7 (11.3 ± 1.3)	8.5–14.3 (12.1 ± 1.2)	5.2–9.6 (7.6 ± 1.3)
Area of nucleus	0.8–1.6 (1.2 ± 0.2)	0.6–1.8 (1.3 ± 0.3)	0.8–1.7 (1.2 ± 0.3)

^a^All measurements are given in micrometers. Minimum and maximum values are provided, followed in parentheses by the arithmetic mean and standard deviation

### *In vitro* development of *Haemoproteus noctuae* and *H. syrnii*

**Fig. 3:**
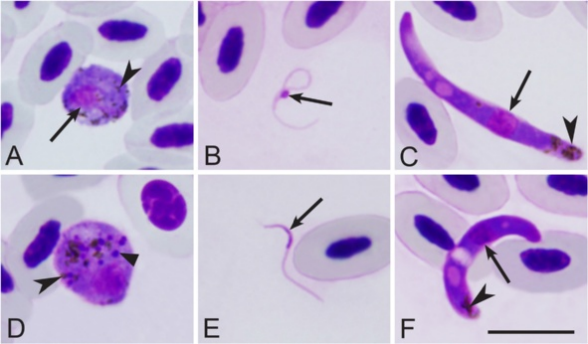
*In vitro* mature gametes and ookinetes of *Haemoproteus noctuae* (lineage hCIRCUM01, **a**–**c**) and *Haemoproteus syrnii* (hCULCIB01, **d**–**f**). Macrogametes (**a**, **d**), microgametes (**b**, **e**) and ookinetes (**c**, **f**) are shown. Long simple arrows – nuclei of parasites, simple arrowheads – pigment granules, triangle arrowheads – volutin granules. Scale bar = 10 μm

Microgametes, macrogametes and ookinetes of *H. noctuae* and *H. syrnii* developed *in vitro* and were observed 12 h after exposure of mature gametocytes to air (Fig. 3a–f). Macrogametes of both species possess prominent pigment granules (Fig. 3a, d). Volutin, which was inherited from macrogametocytes (Fig. 2b), is visible in *H. syrnii* macrogametes (Fig. 3d). Microgametes of both parasites were thread-like bodies possessing readily visible nuclei (Fig. 3b, e). Ookinetes of *H. noctuae* and *H. syrnii* were worm-like bodies possessing prominent nuclei, ‘vacuoles’ and pigment granules (Fig. 3c, f). Volutin granules were invisible in ookinetes of *H. syrnii* (Fig. 3f). Both *H. noctuae* and *H. syrnii* ookinetes, which developed *in vitro,* were morphologically similar to those developed *in vivo* (compare Fig. 2e, f with Fig. 3c, f).

### Phylogenetic analysis

**Fig. 4:**
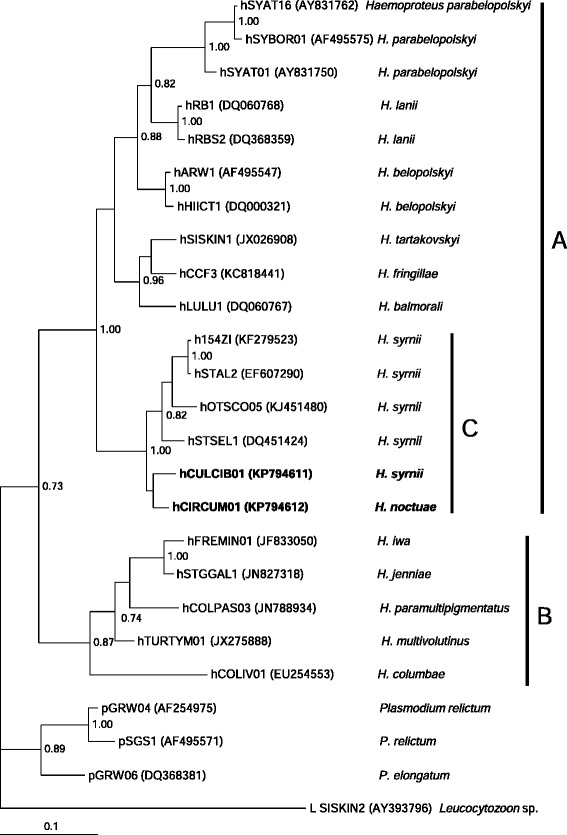
Bayesian phylogeny of 16 *Haemoproteus* (*Parahaemoproteus*) spp., 5 *H.* (*Haemoproteus*) spp. and 3 *Plasmodium* spp. based on cytochrome *b* gene sequences of 479 bp lengths. One *Leucocytozoon* sp. sequence was used as outgroup. Nodal support values indicate Bayesian posterior probabilities. The scale bar shows the expected substitutions per site. Parasite lineage codes are given according to MalAvi database [17]; they are followed by GenBank accession numbers of sequences and parasite species names. Vertical bars (**a**–**c**) indicate groups of closely related species belonging to the subgenus *Parahaemoproteus* (**a**) and *Haemoproteus* (**b**); lineages of owl parasites are marked by bar C. Parasite lineages used in this study are given in bold font

Phylogenetic analysis placed all *Culicoides* spp.-transmitted avian haemoproteids in a well-supported clade A (Fig. 4), which contains *Parahaemoproteus* parasites. One *H. noctuae* lineage and five *H. syrnii* lineages, including those used in this study (Fig. 4, clade C), appeared in the clade A. That is in accordance with our sporogony study, which showed complete sporogonic development of these parasites in *Culicoides* biting midges (Fig. 2e–j). Lineages of hippoboscid-transmitted parasites of the subgenus *Haemoproteus* were placed in a separate well-supported clade B, which is the sister to clade A (Fig. 4).

## Discussion

There are several key results of this study. First, we add *H. noctuae* and *H. syrnii* to the list of *Culicoides* spp.-transmitted haemoproteids, supporting their belonging to the subgenus *Parahaemoproteus* [5, 12, 13]. Both these parasites completed sporogony in *Culicoides* biting midges, with sporozoites reported in salivary glands (Fig. 2g, i, j), indicating that these flies likely are natural vectors, as has been reported for *Parahaemoproteus* species [2, 5, 16]. Several other *Parahaemoproteus* parasites have been shown experimentally to complete sporogony in *Culicoides* spp. These are *H. danilewskii* [47, 48], *H. dolniki* [5], *H. handai* [1], *H. mansoni* [27], *H. nettionis* [49], and *H. velans* [50]. However, molecular characterisation of these avian haemoproteids has not been developed and cyt *b* sequence information is absent, thus they cannot be included in the phylogenetic analysis (Fig. 4).

Second, the phylogenetic analysis placed cyt *b* lineages both of *H. noctuae* and *H. syrnii* in the clade A (Fig. 4). This clade contains nine species of avian *Parahaemoproteus* spp., for which molecular characterization has been developed and sporogony investigated experimentally. These parasites infect passeriform and strigiform birds and complete sporogony in *Culicoides* biting midges [5, 16]. This phylogenetic study (Fig. 4) confirms results of our experiments. In other words, vector studies and phylogenetic analysis based on cyt *b* gene complement each other [5, 16, 51, this study], indicating that the genetic differences in cyt *b* gene between *Parahaemoproteus* and *Haemoproteus* parasites likely reflect differences in their sporogonic stages. Mainly, species of the subgenus *Parahaemoproteus* are characterised by small oocysts (<20 μm in diameter) that possess one germinative centre, a relatively small number of sporozoites in mature oocysts (<100), sporozoites that are usually pointed at both ends, transmitted by biting midges belonging to the Ceratopogonidae, and some other features [5, 13, 52]. Haemoproteids, which are transmitted by hippoboscid flies (Hippoboscidae) belong to the subgenus *Haemoproteus*; they are characterized by large oocysts (>20 μm in diameter) that possess numerous germinative centres, relatively many sporozoites in mature oocysts (>500 sporozoites) that are usually blunt at one end and pointed in the other, and some other features [5, 13, 52]. Based on obtained results (Figs. 2, 3 and 4), we predict that all cyt *b* sequences of haemoproteids transmitted by biting midges will be grouped with the closely related lineages of parasites of the clade A (Fig. 4), and the lineages of hippoboscid transmitted parasites will be clustered with lineages of the clade B (Fig. 4). In other words, the phylogenetic analyses of cyt *b* sequences likely provide opportunities to predict groups of vectors (species of the Ceratopogonidae or Hippoboscidae), which are involved in transmission of avian haemoproteids. Determination of haemosporidian vectors is a time-consuming process, which requires more parasitology research, including infection and dissection of blood-sucking insects and search for sporozoites in salivary glands [11, 16, 27]. That explains why vector species remain unidentified for the great majority of avian *Haemoproteus* and other haemosporidian species, which is particularly true on the level of their numerous genetic lineages. If phylogenies based on cyt *b* gene indicate *Culicoides* spp. vectors, such knowledge will increase vector studies of avian haemoproteids because the methodology of generation of cyt *b* sequence information is relatively inexpensive, easy and well-developed in many laboratories [17–22, 53–59]. We recommend using cyt *b* gene phylogenies for prediction vector groups (Ceratopogonidae or Hippoboscidae) of avian haemoproteids. That will increase the determination of potential vectors. However, the application of sporozoite detection methods is crucial for final identification of vectors due to possible abortive sporogonic development, which PCR-based methods do not read [11].

Third, the available experimental data show that the same species of biting midges can transmit numerous *Parahaemoproteus* species, as is the case with *C. impunctatus*. This biting midge is a competent vector of *H. belopolskyi, H. dolniki, H. parabelopolskyi, H. lanii, H. fringillae, H. balmorali, H. noctuae* and *H. syrnii* [5,16,this study]. Additional examples are *H. danilewskii, H. fringillae. H. mansoni* and *H. velans*; these parasites complete sporogony in the biting midge *Culicoides sphagnumensis* [5]. Interestingly, Atkinson [27] proved experimentally the low vector specificity of *Haemoproteus mansoni* (probable synonym is *H. meleagridis*), which completes sporogony in five species of *Culicoides* biting midges. Lack of the strict specialisation in some *Haemoproteus* parasites to complete sporogony in certain species of biting midges and the broad distribution of many species of birds can explain wide geographic distribution of many lineages of avian haemoproteids [17, 18, 21, 54, 58].

It is worth mentioning that morphology of sporogonic stages of *H. noctuae* remains the same during development in different species of biting midges, indication that some morphological characteristics of sporogonic stages can be applied in taxonomic research on species level of haemoproteids. Ookinetes of many *Parahaemoproteus* species can be distinguished morphologically [5, 59]. This study shows that *H. noctuae* and *H. syrnii* can be readily distinguished due to the different lengths of their sporozoites (Table 1), providing an opportunity to distinguish these parasites at sporozoite stage in vectors. Importantly, the length of *H. noctuae* sporozoites was the same during development in *C. impunctatus* and *C. nubeculosus*, supporting the taxonomic value of this character. Presence of unique pedunculated oocysts in chicken malaria parasite *Plasmodium juxtanucleare* [60] shows that oocyst morphology is also worth attention in wildlife haemosporidian research. Additional studies are needed for determining taxonomic value of sporogonic stages, which have been insufficiently applied in classification and species identification of haemosporidian parasites.

A recent study [33], which is based on observation of single louse fly *Ornithomyia* sp. sampled on one tawny owl, speculated that *H. syrnii* might be transmitted by this hippoboscid fly. These authors observed and illustrated gametocytes and ookinetes of *H. syrn*ii in a preparation made from the naturally infected fly, which was squashed on a glass slide. However, the observation of these parasite stages in the fly does not necessarily indicate competent vector. When blood-sucking insects take blood meal on an infected bird, mature gametocytes are ingested. The latter readily produce gametes, and ookinetes develop in non-vector blood-sucking insects and even *in vitro* [5, 59]. Our study shows that both *H. noctuae* and *H. syrnii* readily exflagellate and produce ookinetes *in vitro* (Fig. 3). Thus, it is not unexpected to observe them in engorged hippoboscids. Demonstration of sporozoites in salivary glands is an essential step for definitive demonstration that insects can act as vectors [11, 16, 24, 27, 49]. These authors [33] believed that they observed sporozoites in this fly, but provided illustration of a massive bacterial infection, which they attributed to sporozoites (Figures 32–34 in [33]). Similar bacterial infection has been seen in preparations of blood-sucking dipteran insects, where entire bodies were squashed on slides (G. Valkiūnas, personal observation). Because our experiments provided evidence about complete sporogony both of *H. syrnii* and *H. noctuae* in two species of *Culicoides* biting midges (Fig. 2), we question conclusions about vectorial capacity of hippoboscid flies in transmission of haemoproteids of owls. It seems unlikely that the same *Haemoproteus* sp. could be transmitted both by ceratopogonids and hippoboscids because transmission of the same haemosporidian species by vectors belonging to different families of dipteran insects has not been reported in haemosporidian parasites [5, 22, 52], however this hypothesis requires experimental testing [27].

## Conclusions

This study adds two species of avian haemoproteids to the list of parasites transmitted by biting midges belonging to *Culicoides*. We show that widespread owl haemoproteids *H. noctuae* and *H. syrnii* complete sporogony in the biting midges *C. impunctatus* and *C. nubeculosus*, which are widespread in Europe and are likely to be the natural vectors of these infections. Both parasite species are characterised by rapid sporogony at relatively low temperature; that probably contributes to their spread in countries not only with warm, but also cold climates. We report that morphology of sporozoites of some *Parahaemoproteus* species remains the same when parasite develops into different species of biting midges, but morphology of sporozoites differs in different parasite species during their development in the same species of midges. Thus, morphology of sporozoites can be applied in some *Parahaemoproteus* parasite identification in vectors. This study shows that phylogenies based on cyt *b* gene indicate parasite-vector relationships, and we recommend using them in planning research on avian haemoproteid vectors in wildlife.
